# Long Noncoding RNA MIR100HG Knockdown Attenuates Hepatocellular Carcinoma Progression by Regulating MicroRNA-146b-5p/Chromobox 6

**DOI:** 10.1155/2021/6832518

**Published:** 2021-07-30

**Authors:** Fushun Li, Xianghua Sun, Qing Liu, Xilu Liu, Jia Zhang

**Affiliations:** ^1^Department of Hepatobiliary Surgery, Wei Fang Yi Du Central Hospital, No. 4318, South Linglong Mountain Road, Qingzhou County, Weifang City, Shandong Province 262500, China; ^2^Health Care Department I, Weifang People's Hospital, No. 151, Guangwen Street, Kuiwen District, Weifang City, Shandong Province 261041, China

## Abstract

**Purpose:**

Hepatocellular carcinoma (HCC) accounts for approximately ninety percent of primary liver cancer. This study attempted to investigate the effects of the long noncoding RNA MIR100HG (MIR100HG) in HCC and the underlying molecular mechanism.

**Materials and Methods:**

qRT-PCR was implemented to analyze the expression of MIR100HG, microRNA-146b-5p (miR-146b-5p), and Chromobox 6 (CBX6). The correlation between MIR100HG and clinicopathological features of HCC patients was assessed. Additionally, the effects of MIR100HG knockdown on HCC cell viability, migration, and invasion were explored. The interactions among MIR100HG, miR-146b-5p, and CBX6 were confirmed. Furthermore, rescue experiments were conducted to investigate whether MIR100HG knockdown modulates HCC cell behaviors through modulating the miR-146b-5p/CBX6 axis.

**Results:**

The expression of MIR100HG and CBX6 was enhanced, while miR-146b-5p was inhibited in HCC cells. High MIR100HG expression was positively associated with the TNM tumor stage and Edmondson-Steiner grading in HCC patients. MIR100HG knockdown considerably reduced the HCC cell viability, migration, and invasion. In addition, MIR100HG directly targeted miR-146b-5p, and miR-146b-5p directly targeted CBX6 in HCC cells. Moreover, miR-146b-5p suppression or CBX6 elevation evidently rescued the suppressed viability, migration, and invasion of HCC cells caused by MIR100HG knockdown.

**Conclusions:**

Knockdown of MIR100HG inhibited the viability, migration, and invasion of HCC cells by targeting the miR-146b-5p/CBX6 axis, offering a potential therapeutic target for HCC therapy.

## 1. Introduction

Primary liver cancer is the fourth most common reason of cancer mortality [1]. Hepatocellular carcinoma (HCC) is a frequently malignant tumor of primary liver cancer [2]. HCC is usually accompanied by cirrhosis and chronic viral hepatitis B or C [3]. Despite advances in the clinical treatment of HCC, the prognosis for HCC patients is still poor [4]. Thus, it is crucial to discover new therapeutic targets for HCC treatment.

Emerging evidences reveal that long noncoding RNAs (lncRNAs) are involved in the regulation of cell proliferation, migration, and invasion in several types of human cancers, such as osteosarcoma [5], breast cancer [6], and lung cancer [7]. Surely, some lncRNAs acting as oncogenes are also reported to participate in HCC tumorigenesis. lncRNA FAL1 inhibits miR-1236 expression to promote HCC cell proliferation and metastasis [8]. lncRNA KTN1-S1 silencing reduces viability and triggers apoptosis of HCC cells [9]. lncRNA MALAT1 contributes to HCC progression via mTOR activation and SRSF1 elevation [10]. It has been documented that lncRNA MIR100HG (MIR100HG) serves as an oncogene to take part in solid tumors. MIR100HG elevation accelerates the metastasis of colorectal cancer and is related to poor outcome [11]. MIR100HG overexpression accelerates the laryngeal squamous cell carcinoma progression by inhibiting miR-204-5p [12]. Elimination of MIR100HG attenuates the cell proliferation in gastric cancer [13]. However, the specific regulatory function of MIR100HG in HCC remains unknown.

Certain microRNAs (miRNAs) have been determined to participate in HCC. miR-200b overexpression inhibits HCC cell growth via suppressing HMGB3 expression [14]. miR-490-5p represses the ECT2 and E2F2 expression to attenuate metastasis of HCC [15]. miR-136 regulates the COX2 expression, thus restraining the malignant progression of HCC cells [16]. miR-146b-5p acts as an anti-oncomiR in diverse cancers. miR-146b-5p elevation inhibits NOTCH1 to decrease T-cell acute lymphoblastic leukemia progression [17]. miR-146b-5p deficiency enhances glioma cell growth while it attenuates apoptosis by regulating TRAF6 [18]. Notably, miR-146b-5p exerts the tumor inhibitory effect by retarding the phosphorylation of Akt in HCC [19]. However, the regulatory relationship between MIR100HG and miR-146b-5p in HCC remains unclear.

Chromobox 6 (CBX6), one of the polycomb paralogs, functions in cancer progression [20]. Certain CBX family members exert a tumor-promoting role in HCC. CBX8 interacts with YBX1 to accelerate HCC cell proliferation [21]. CBX4 silencing impairs cell cycle progression and hampers cell proliferation in HCC [22]. Importantly, CBX6 overexpression is an independent prognostic factor for shorter overall survival of HCC patients [23]. However, the regulatory relationship between MIR100HG and CBX6 in HCC remains to be elucidated.

Herein, we evaluated the expression and functions of MIR100HG in HCC. Additionally, the relationships between MIR100HG, miR-146b-5p, and CBX6 in HCC were confirmed. This study may provide a novel therapeutic target for HCC.

## 2. Material and Method

### 2.1. Human Samples

Fifty HCC patients who underwent hepatectomy from June 2017 to March 2019 at our hospital were enrolled in this study. Fifty HCC tissue specimens (tumor group) and paired adjacent tissues (normal group) were obtained from the HCC patients who underwent hepatectomy. Prior to hepatectomy, no radiotherapy or chemotherapy treatment was administered to the patients. The patient cohort was separated into high MIR100HG (*n* = 25) and low MIR100HG groups (*n* = 25) according to the median MIR100HG expression. This study was permitted by our hospital ethics committee, and informed consent was obtained from each patient.

### 2.2. Cell Culture

The Hep3B, HepG2, SK-HEP1, and Huh7 HCC cell lines and normal LO2 liver cell line (American Type Culture Collection, Manassas VA, USA) were cultured in Dulbecco's modified Eagle's medium (DMEM, Invitrogen, Carlsbad, CA, USA) supplied with 10% fetal bovine serum (FBS) at 37°C with 5% CO_2_.

### 2.3. Cell Transfection

The small interfering (si) MIR100HG-1, si-MIR100HG-2, si-negative control (NC), miR-146b-5p mimics, mimic NC, miR-146b-5p inhibitor, inhibitor NC, pcDNA3.1 (pcDNA)-CBX6, and pcDNA-NC were synthesized by GenePharma (Shanghai, China). Hep3B and SK-HEP1 cells grown to 80% confluence were transfected or cotransfected with these above agents using the Lipofectamine 3000 reagent (Invitrogen). Forty-eight hours posttransfection, Hep3B and SK-HEP1 cells were used for further assays.

### 2.4. Quantitative Real-Time Polymerase Chain Reaction (qRT-PCR) and Western Blot

qRT-PCR and western blot were performed as previously described [24]. The primers are depicted in Table 1. MIR100HG, miR-146b-5p, and CBX6 expression was normalized to GAPDH, U6, and *β*-actin expression. The antibodies for western blot analysis including anti-CBX6 (1 : 1000, av39074, Sigma, St. Louis, MO, USA), anti-GAPDH (1 : 1000, G9545, Sigma), and HRP-conjugated secondary antibody (1 : 2000, 12-348, Sigma). The protein bands were visualized by enhanced chemiluminescence exposure solution and quantified by ImageLab software (Bio-Rad, Hercules, CA, USA).

### 2.5. MTT Assay

Hep3B and SK-HEP1 cells (2 × 10^3^/well) were seeded into 96-well plates and incubated at 37°C with 5% CO_2_. At each time point (0, 24, 48, and 72 h posttransfection), cell viability was measured via the MTT cell viability assay kit (Sigma) by the manufacturer.

### 2.6. Wound Healing Assay

Hep3B and SK-HEP1 cells (1 × 10^6^/well) were incubated in 6-well plates. The cell monolayer was then wounded with a 10 *μ*l pipette tip and cultured in serum-free medium. Cell migration images were captured at 0 and 24 h.

### 2.7. Invasion Assay

A Transwell chamber (8 mm, Corning Incorporated, Corning, NY, USA) was used to measure cell invasion. Briefly, Hep3B and SK-HEP1 cells (2.5 × 10^5^/well) in serum-free medium were added into the upper chamber. Medium containing 10% FBS was added to the lower chamber and acted as a chemoattractant. After 24 h, cells that had invaded the pore were fixed with methanol and stained with 0.05% crystal purple. The invading cells were counted.

### 2.8. Dual-Luciferase Reporter Assay

The potential binding sites of MIR100HG and miR-146b-5p or miR-146b-5p and CBX6 were predicted by starBase or TargetScan, respectively. We generated MIR100HG and CBX6 sequences with WT or MUT miR-146b-5p-binding sites and cloned them in the pmirGLO vector (YouBio, Hunan, China). Hep3B and SK-HEP1 cells were cotransfected with above luciferase vectors and mimic NC or miR-146b-5p mimics using Lipofectamine 3000 (Invitrogen).

### 2.9. Statistical Analyses

All statistical analyses were performed using GraphPad Prism (version 8.0). Data are presented as the mean ± SD. The differences between two groups or among multiple groups were assessed by Student's *t*-test or one-way ANOVA followed by Tukey's post hoc test. The significance of the correlations was determined by Pearson's correlation analysis. *P* values < 0.05 were considered statistically significant.

## 3. Results

### 3.1. MIR100HG Expression Enhanced in HCC

**Figure 1:**
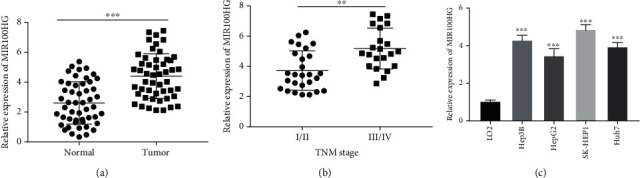
The expression of MIR100HG was enhanced in hepatocellular carcinoma (HCC) tissues and cells. (a) The expression of MIR100HG in HCC tumor tissues and adjacent tissues was detected by qRT-PCR. ^∗∗∗^*P* < 0.001 vs. normal. (b) Relative expression of MIR100HG in HCC patients at the TNM I/II and TNM III/IV. ^∗∗^*P* < 0.01 vs. I/II. (c) qRT-PCR was performed to measure the expression of MIR100HG in LO2, Hep3B, HepG2, SK-HEP1, and Huh7 cells. ^∗∗∗^*P* < 0.001 vs. LO2.

Figure 1(a) exhibits that MIR100HG expression was considerably increased in tumor tissues of HCC patients (*P* < 0.001). Besides, MIR100HG expression was clearly enhanced in tumors at TNM III/IV (*P* < 0.01, Figure 1(b)). As depicted in Table 2, increased MIR100HG expression was related to the TNM tumor stage and Edmondson-Steiner grading in HCC patients (*P* < 0.05). Moreover, MIR100HG expression was dramatically upregulated in Hep3B, HepG2, SK-HEP1, and Huh7 cells compared to that in LO2 cells (*P* < 0.001, Figure 1(c)).

### 3.2. MIR100HG Knockdown Attenuated the Tumorigenesis of HCC Cells

**Figure 2:**
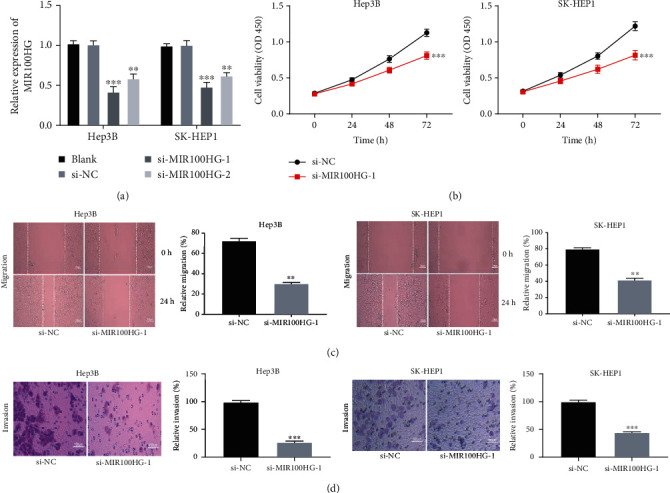
MIR100HG knockdown attenuated the tumorigenesis of hepatocellular carcinoma (HCC) cells. (a) The transfection efficiency of si-NC, si-MIR100HG-1, and si-MIR100HG-2 in Hep3B and SK-HEP1 cells was evaluated by qRT-PCR. ^∗∗^*P* < 0.01, ^∗∗∗^*P* < 0.001 vs. si-NC. (b) The viability of Hep3B and SK-HEP1 cells was measured by the MTT assay. ^∗∗∗^*P* < 0.001 vs. si-NC. (c, d) The migration and invasion of Hep3B and SK-HEP1 cells were determined by the wound healing assay and invasion assay. ^∗∗^*P* < 0.01, ^∗∗∗^*P* < 0.001 vs. si-NC.

MIR100HG was blocked by transfecting with si-MIR100HG-1 (*P* < 0.001) and si-MIR100HG-2 (*P* < 0.01) in Hep3B and SK-HEP1 cells (Figure 2(a)). si-MIR100HG-1 was used for subsequent assays because of high silence efficiency. The MTT assay demonstrated that si-MIR100HG-1 markedly reduced the viability of Hep3B and SK-HEP1 cells (*P* < 0.001, Figure 2(b)). As illustrated in Figures 2(c) and 2(d), the migration and invasion of Hep3B and SK-HEP1 cells were visibly inhibited following MIR100HG knockdown (*P* < 0.01).

### 3.3. miR-146b-5p Was Directly Targeted by MIR100HG

**Figure 3:**
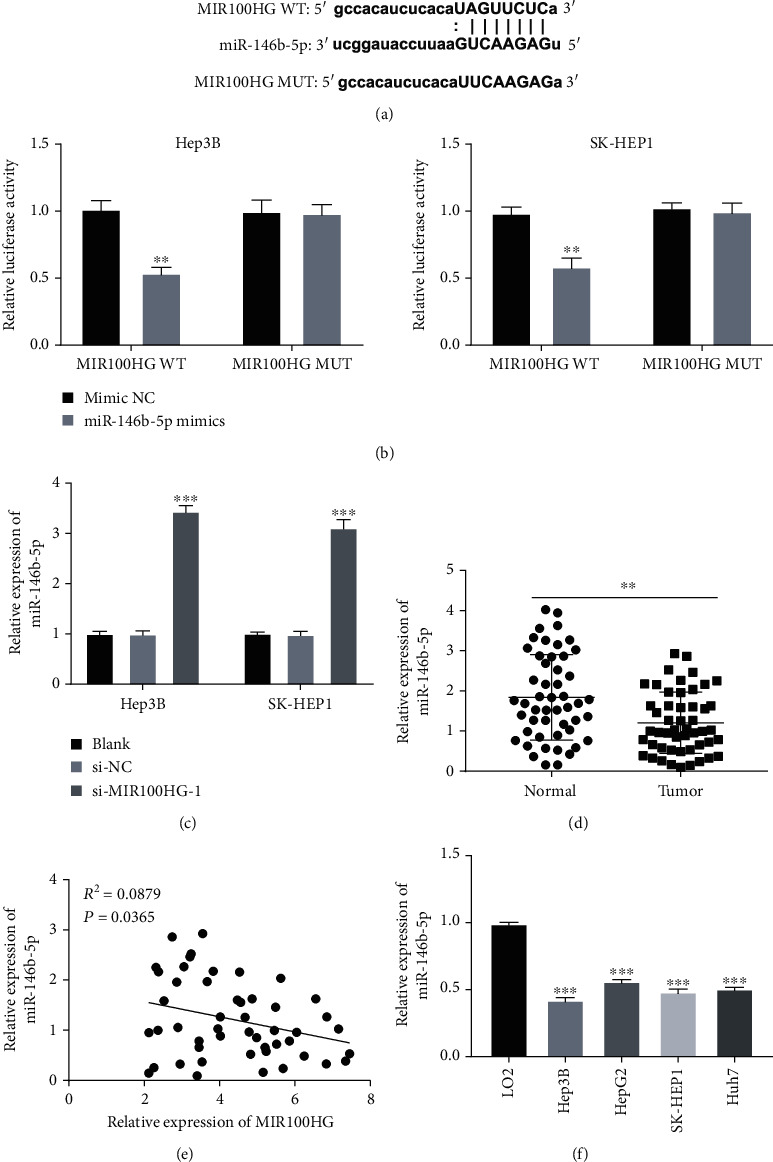
miR-146b-5p was directly targeted by MIR100HG. (a) starBase displayed the predicted binding site between MIR100HG and miR-146b-5p. (b) Relative luciferase activity in Hep3B and SK-HEP1 cells was measured by the dual-luciferase reporter assay. ^∗∗^*P* < 0.01 vs. mimic NC. (c) The expression of miR-146b-5p was upregulated by the transfection of si-MIR100HG-1 in Hep3B and SK-HEP1 cells. ^∗∗∗^*P* < 0.001 vs. si-NC. (d) qRT-PCR was performed to determine the expression of miR-146b-5p in hepatocellular carcinoma (HCC) tumor tissues and adjacent tissues. ^∗∗^*P* < 0.01 vs. normal. (e) The expression of MIR100HG was negatively correlated with miR-146b-5p in HCC tumor tissues. (f) The expression of miR-146b-5p in LO2, Hep3B, HepG2, SK-HEP1, and Huh7 cells was detected by qRT-PCR. ^∗∗∗^*P* < 0.001 vs. LO2.

Bioinformatics analysis was performed with StarBase to predict that MIR100HG directly targets miR-146b-5p (Figure 3(a)). To demonstrate this prediction, the dual-luciferase reporter assay was used, and the results discovered that fluorescence intensity was clearly constrained in Hep3B and SK-HEP1 cells after transfecting with MIR100HG 3′UTR-WT and miR-146b-5p mimics (*P* < 0.01, Figure 3(b)). MIR100HG knockdown could markedly upregulate miR-146b-5p expression in Hep3B and SK-HEP1 cells (*P* < 0.001, Figure 3(c)). Additionally, miR-146b-5p expression was considerably suppressed in tumor tissues of HCC patients (*P* < 0.01, Figure 3(d)). Interestingly, MIR100HG and miR-146b-5p expression was negatively correlated in tumor tissues (*R*^2^ = 0.0879, *P* = 0.0365, Figure 3(e)). miR-146b-5p expression was clearly inhibited in Hep3B, HepG2, SK-HEP1, and Huh7 cells compared with that in LO2 cells (*P* < 0.001, Figure 3(f)).

### 3.4. miR-146b-5p Restrained the Tumorigenesis of HCC Cells

**Figure 4:**
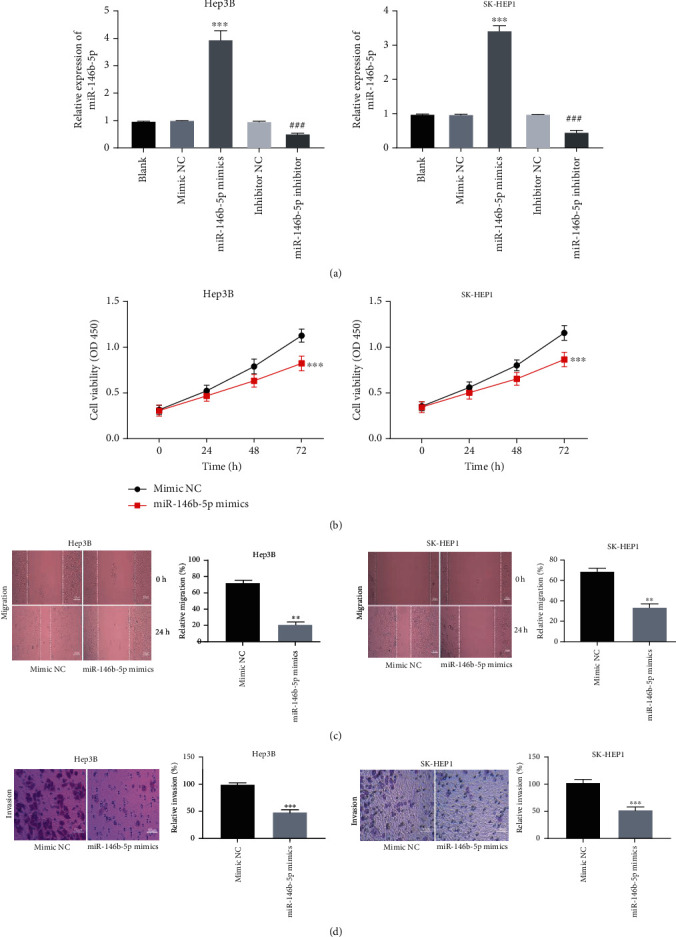
miR-146b-5p restrained the tumorigenesis of hepatocellular carcinoma (HCC) cells. (a) The transfection efficiency of mimic NC, miR-146b-5p mimics, inhibitor NC, and miR-146b-5p inhibitor was measured by qRT-PCR in Hep3B and SK-HEP1 cells. ^∗∗∗^*P* < 0.001 vs. mimic NC, ^###^*P* < 0.001 vs. inhibitor NC. (b) MTT assay was performed after transfection with mimic NC or miR-146b-5p mimics in Hep3B and SK-HEP1 cells. ^∗∗∗^*P* < 0.001 vs. mimic NC. (c, d) The effects of miR-146b-5p elevation on the migration and invasion of Hep3B and SK-HEP1 cells were evaluated. ^∗∗^*P* < 0.01, ^∗∗∗^*P* < 0.001 vs. mimic NC.

miR-146b-5p was elevated or inhibited by transfecting with miR-146b-5p mimics or miR-146b-5p inhibitor in Hep3B and SK-HEP1 cells (*P* < 0.001, Figure 4(a)). miR-146b-5p elevation strikingly attenuated the viability of Hep3B and SK-HEP1 cells (*P* < 0.001, Figure 4(b)). Beside, miR-146b-5p significantly impeded the migration and invasion of Hep3B and SK-HEP1 cells (*P* < 0.01, Figures 4(c) and 4(d)).

### 3.5. miR-146b-5p Directly Targeted CBX6

**Figure 5:**
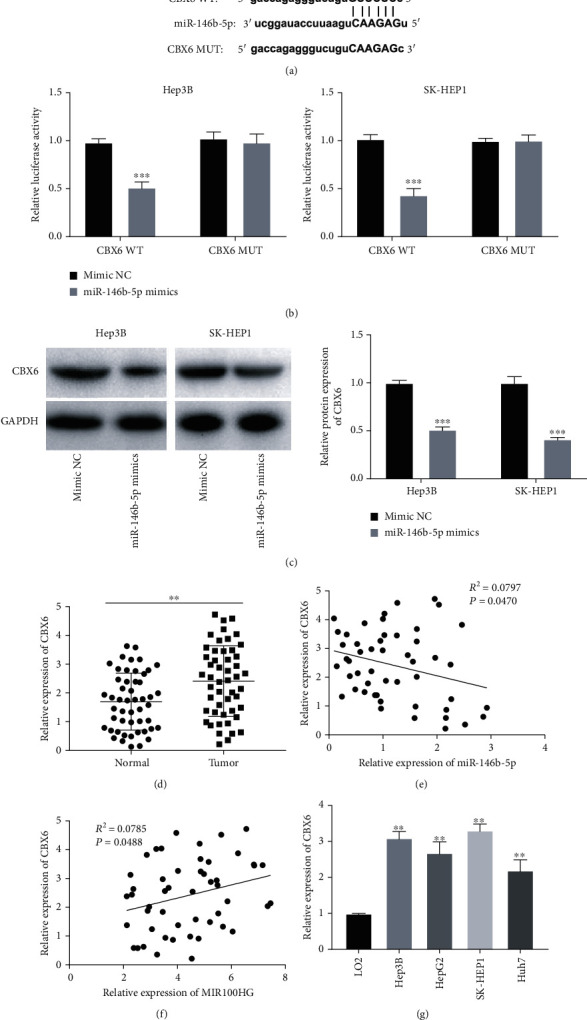
miR-146b-5p directly targeted CBX6. (a) TargetScan exhibited the predicted binding site between CBX6 and miR-146b-5p. (b) Dual-luciferase reporter assay was performed to measure the relative luciferase activity in Hep3B and SK-HEP1 cells. ^∗∗∗^*P* < 0.001 vs. mimic NC. (c) The protein expression of CBX6 in Hep3B and SK-HEP1 cells was measured by western blot. ^∗∗∗^*P* < 0.001 vs. mimic NC. (d) qRT-PCR was used to detect the expression of CBX6 in hepatocellular carcinoma (HCC) tumor tissues and adjacent tissues. ^∗∗^*P* < 0.01 vs. normal. (e) The expression of CBX6 was negatively correlated with miR-146b-5p in HCC tissues. (f) The expression of CBX6 was positively correlated with MIR100HG in HCC tissues. (g) qRT-PCR was performed to evaluate the expression of CBX6 in LO2, Hep3B, HepG2, SK-HEP1, and Huh7 cells. ^∗∗^*P* < 0.01 vs. LO2.

The binding site of miR-146b-5p, as predicted by TargetScan, was at the 3′UTR of CBX6 (Figure 5(a)). The dual-luciferase reporter assay revealed that fluorescence intensity was visibly suppressed in Hep3B and SK-HEP1 cells after being transfected with CBX6 3′UTR-WT and miR-146b-5p mimics (*P* < 0.001, Figure 5(b)). Besides, miR-146b-5p elevation obviously repressed the CBX6 protein expression in Hep3B and SK-HEP1 cells (*P* < 0.001, Figure 5(c)). Moreover, CBX6 expression was dramatically elevated in tumor tissues of HCC patients (*P* < 0.01, Figure 5(d)). There were a negative correlation between CBX6 and miR-146b-5p expression (*R*^2^ = 0.0797, *P* = 0.0470, Figure 5(e)) and a positive correlation between CBX6 and MIR100HG expression in HCC tissues (*R*^2^ = 0.0785, *P* = 0.0488, Figure 5(f)). CBX6 expression was visibly increased in Hep3B, HepG2, SK-HEP1, and Huh7 cells compared to that in LO2 cells (*P* < 0.01, Figure 5(g)).

### 3.6. MIR100HG Deficiency Suppressed the Tumorigenesis of HCC Cells by Targeting the miR-146b-5p/CBX6 Axis

**Figure 6:**
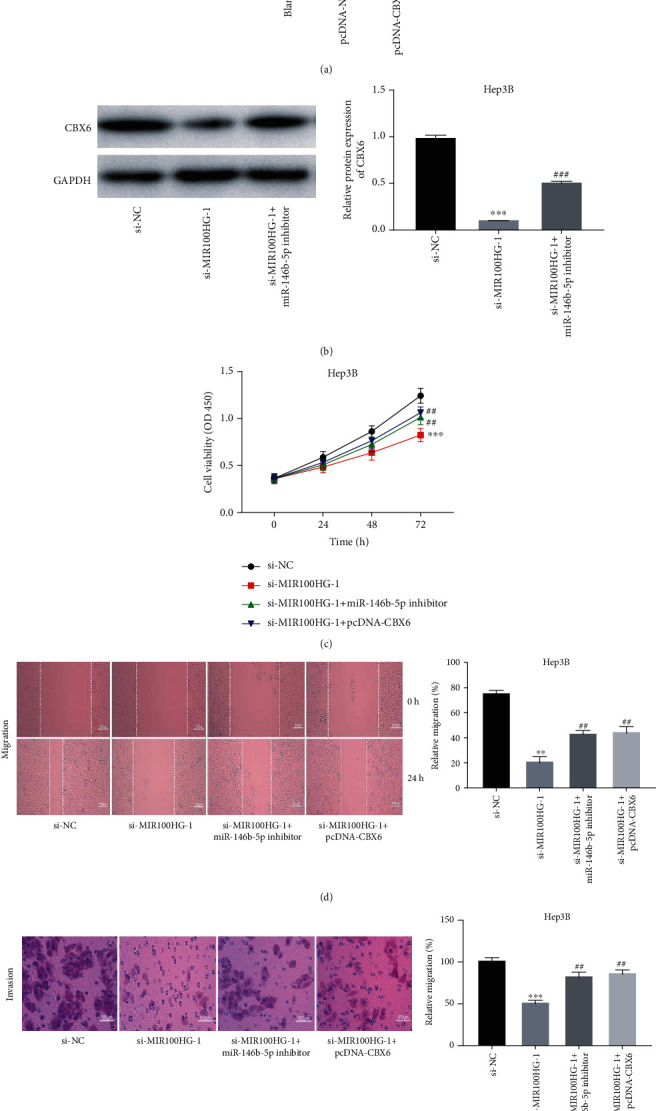
MIR100HG deficiency suppressed the tumorigenesis of hepatocellular carcinoma (HCC) cells by targeting the miR-146b-5p/CBX6 axis. (a) The transfection efficiency of pcDNA-NC and pcDNA-CBX6 was measured by qRT-PCR in Hep3B cells. ^∗∗∗^*P* < 0.001 vs. pcDNA-NC. (b) Western blot was performed to measure the protein expression of CBX6 in Hep3B cells. ^∗∗∗^*P* < 0.001 vs. si-NC, ^###^*P* < 0.001 vs. si-MIR100HG-1. (c–e) Inhibition of miR-146b-5p or overexpression of CBX6 reversed the inhibitory effects of MIR100HG knockdown on viability, migration, and invasion of Hep3B cells. ^∗∗^*P* < 0.01, ^∗∗∗^*P* < 0.001 vs. si-NC; ^##^*P* < 0.01 vs. si-MIR100HG-1.

Figure 6(a) displays that CBX6 expression was clearly elevated by transfecting with pcDNA-CBX6 in Hep3B cells (*P* < 0.001). Decreased CBX6 protein expression by MIR100HG knockdown in Hep3B cells was rescued by miR-146b-5p suppression (*P* < 0.001, Figure 6(b)). We further explored the molecular mechanism by which MIR100HG knockdown inhibited the tumorigenesis of HCC cells, using rescue experiments. As exhibited in Figures 6(c) – 6(e), miR-146b-5p elimination or CBX6 overexpression evidently prevented the inhibitory effects of MIR100HG suppression on Hep3B cell viability, migration, and invasion (*P* < 0.01).

## 4. Discussion

The lncRNA elevation often facilitates HCC progression [25]. The expression of lncRNAs, such as MALAT1 [10], AB019562 [24], and CARLo-5 [26], is enhanced in HCC patients. In the present research, MIR100HG expression was elevated in HCC, indicating that MIR100HG may be an oncogenic lncRNA in HCC. Besides, MIR100HG expression was found to be correlated with the TNM tumor stage and Edmondson-Steiner grading in HCC patients. Some lncRNAs have the same function in HCC. lncRNA CARLo-5 is upregulated in HCC patients with a higher Edmondson-Steiner grade [26]. High-level lncRNA LINC00470 is markedly correlated with an advanced TNM stage in HCC patients [27]. Above all, MIR100HG expression was enhanced in HCC and related to the HCC progression. Numerous researches have demonstrated that MIR100HG partakes in tumor behavior of different cancers [11, 12]. Knockdown of MIR100HG has an inhibitory effect on the tumorigenesis of cancers. For instances, MIR100HG suppression impairs the cell viability, thus attenuating the tumorigenesis of acute megakaryoblastic leukemia [28]. MIR100HG deficiency impedes cell viability while accelerating cell apoptosis to suppress HCC progression [29]. MIR100HG silencing upregulates miR-5590-3p and downregulates OTX1, resulting in the decrease in cell viability and invasion in breast cancer [30]. In this study, MIR100HG knockdown decreases the HCC cell viability, invasion, and migration, suggesting that MIR100HG acts as a therapeutic target for HCC treatment. In addition, sorafenib, an oral kinase inhibitor, has been confirmed to be a gold quality standard to enhance survival in patients affected by advanced HCC in the first-line treatment [31]. Interestingly, numerous lncRNAs are overexpressed in HCC tissues and are closely associated with sorafenib resistance, such as SNHG1 [32], SNHG3 [33], HEIH [34], and NEAT1 [35]. We speculated that the high expression level of MIR100HG may also promote sorafenib resistance to affect the progression of HCC when sorafenib is administered in clinical practice. Further researches on the interaction of MIR100HG with sorafenib are urgently needed.

lncRNAs often function as a molecular sponge or competing endogenous RNA in regulating miRNA in HCC. For instances, lncRNA n335586 facilitates CKMT1A expression through competitively binding with miR-924 to enhance HCC cell migration and invasion [36]. lncRNA SNHG15 serves as a molecular sponge of miR-141-3p to exert its oncogenic effect in HCC [37]. Notably, lncRNA MALAT1 accelerates HCC progression via sponging miR-146b-5p [19]. Here, miR-146b-5p was a target of MIR100HG and negatively related to MIR100HG expression, indicating that MIR100HG may be involved in HCC by regulating miR-146b-5p. miR-146b-5p is often inhibited in solid tumors, such as glioma [38] and gallbladder cancer [39]. In this study, miR-146b-5p was suppressed in HCC, suggesting that miR-146b-5p may be an anti-oncomiR in HCC. It has been documented that miR-146b-5p serves as a mediator in tumor progression. miR-146b-5p upregulation leads to MMP16 knockdown, thus hindering glioma cell growth [38]. miR-146b-5p overexpression retards cell proliferation and facilitates cell apoptosis in nasopharyngeal carcinoma by regulating HNRNPA2B1 [40]. Importantly, miR-146b-5p is inhibited in HCC cells and is a biomarker for the gene therapy of HCC [41]. Here, we determined that miR-146b-5p restrained tumorigenesis of HCC cells, and miR-146b-5p deficiency weakened the antitumor function of MIR100HG suppression in HCC cells. Taken together, MIR100HG silencing may inhibit tumorigenesis of HCC cells through upregulating miR-146b-5p.

CBX family members are frequently elevated in various tumors, such as CBX2 in ovarian cancer [42], CBX4 in breast cancer [43],, and CBX8 in muscle invasive bladder cancer [44]. Similarly, CBX6 expression was increased in HCC in this study, indicating that CBX6 may be a participant in HCC. CBX family numbers are involved in HCC progression. CBX1 interacts with HMGA2 to accelerate HCC cell proliferation and migration [45]. CBX2 suppression attenuates proliferation and induces apoptosis in HCC cells by modulating YAP [46]. Notably, CBX6 elevation promotes HCC cell growth and is predictive of a poor outcome in HCC [47]. Additionally, the CBX family member can be involved in HCC progression as a target gene for miRNAs. For example, miR-195 overexpression restrains proliferative and invasive capacities of the HCC cells by inhibiting CBX4 [48]. In the present study, CBX6 was demonstrated to be a target of miR-146b-5p, which was inversely modulated through miR-146b-5p. We assumed that miR-146b-5p may suppress HCC progression through inhibiting CBX6. Furthermore, we discovered that CBX6 expression was positively related to MIR100HG in HCC. Considering the MIR100HG/miR-146b-5p axis, we speculated that MIR100HG may influence CBX6 via modulating miR-146b-5p. Importantly, rescue experiment exhibited that CBX6 overexpression reversed the suppressed HCC cell behaviors caused by MIR100HG silencing. To sum up, MIR100HG silencing may attenuate the tumorigenesis of HCC cells by regulating the miR-146b-5p/CBX6 axis. Additionally, the expression of MIR100HG/miR-146b-5p/CBX6 in HCC tissues was based on hepatectomy in this study. In many cases, however, biomarkers are not uniformly present in all cancer cells, and such heterogeneity might hinder the therapeutic efficacy of tailored therapies [49]. In recent years, there has been an increasing development of liquid biopsy to replace tissue biopsy [50]. Blood-based liquid biopsy consists in the isolation and analysis of tumor-derived or tumor-associated components that circulate in the bloodstream, including circulating tumor cells (CTCs), circulating leukocytes, and tumor-derived circulating nucleic acids, such as cell-free circulating tumor DNA (ctDNA), miRNA, and lncRNAs [51, 52]. In fact, these components are deeply involved in the epithelial-to-mesenchymal transition (EMT) process of HCC, which greatly affect cell invasion and migration [53–55]. We speculated that if so, the measurement of MIR100HG/miR-146b-5p/CBX6 expression by liquid biopsy may be a more efficient and rapid method to determine whether the metastasis occurs in HCC patients. This may be also a limitation of this study, and we will consider this in future studies. Even so, our findings for the first time uncover new insight for the molecular mechanism of HCC and provide a potential target for HCC treatment.

## Figures and Tables

**Table 1:** Primer sequences.

Name of primer	Sequences (5′-3′)
MIR100HG-F	CCCAGTGCAAGGACAAAGA
MIR100HG-R	GCAGAGGAGGTGTCTTCAGG
GAPDH-F	GGGAAATTCAACGGCACAGT
GAPDH-R	AGATGGTGATGGGCTTCCC
miR-146b-5p-F	ACACTCCAGCTGGGTGAGAACTGAATTCCA
miR-146b-5p-R	TGGTGTCGTGGAGTCGGCAATT
U6-F	CTCGCTTCGGCAGCACA
U6-R	AACGCTTCACGAATTTGCGT
CBX6-F	AGATGTCACCCTGCTCCAAT
CBX6-R	AGCCACCTTCTCGAAATCCT
*β*-Actin-F	AGAAAATCTGGCACCACACC
*β*-Actin-R	AGAGGCGTACAGGGATAGCA

**Table 2:** Correlation between MIR100HG expression and clinicopathological features in hepatocellular carcinoma patients.

Characteristics	*n*	MIR100HG		*P* value
		Low (*n* = 25)	High (*n* = 25)	
Age				0.921
<50 years	24	11	13	
≥50 years	26	14	12	

Gender				0.382
Male	29	16	13	
Females	21	9	12	

Liver cirrhosis				0.261
Yes	38	20	18	
No	12	5	7	

Tumor size				0.428
<5 cm	32	18	14	
≥5 cm	18	7	11	

TNM tumor stage				0.034^∗^
I+II	28	19	9	
III+IV	22	6	16	

Edmondson-Steiner grading				0.046^∗^
I+II	31	21	10	
III+IV	19	4	15	

Note: ^∗^*P* < 0.05.

## Data Availability

All data are available through the responsible corresponding author.
